# Comparative evaluation of a new frugal binocular indirect ophthalmoscope

**DOI:** 10.1038/s41433-021-01901-7

**Published:** 2021-12-23

**Authors:** Obaid Kousha, Sharma Ganesananthan, Bayan Shahin, John Ellis, Andrew Blaikie

**Affiliations:** 1 Infection & Global Health Division, School of Medicine, Medical and Biological Sciences Building, North Haugh, St Andrews, KY16 9TF UK; 2 Ninewells Hospital, NHS Tayside, James Arrott Dr, Dundee, DD2 1SG UK; 3 Queen Margaret Hospital, NHS Fife, Whitefield Road, Dunfermline, KY12 OSU UK

**Keywords:** Retinal diseases, Macular degeneration

## Abstract

**Objectives:**

We compare the optical quality and design characteristic a new low cost solar powered binocular indirect ophthalmoscope (BIO), Holo, to Keeler BIO.

**Methods:**

Twenty-four participants each examined 10 simulation eyes using both the Holo and the Keeler BIO with a 30-diopter condensing lens. Number of Lea symbols printed on the retina of simulation eyes seen and time taken to identify them was recorded. Stereoacuity of 12 participants was tested while using the BIOs. Using 7-point Likert scale, participants gave feedback on design characteristic of both BIOs.

**Results:**

There was no statistical difference in number of Lea symbols correctly identified (15.63/20 for Holo vs. 15/20 for Keeler BIO, *p* = 0.366, paired *t* test) or time taken to correctly identify each symbol (Holo 0.39 s faster; 95% confidence interval −2.24 to 3.03 s, *p* = 0.763) using each device. 12 out of 12 participants achieved stereoacuity of 60 arcsec using the Holo while with the Keeler BIO 11 achieved 60 arcsec and one 90 arcsec. There was no statistically significant difference in the scores for clarity of view, quality of illumination, field of view, binocularity, eye strain and robustness between the two devices. The Holo, scored higher for ease of use (6.5 vs. 6, *p* = 0.00488, Wilcoxon signed-rank test), comfort of wear (6 vs. 5, *p* = 0.000337) and portability (7 vs. 6, *p* = 0.000148).

**Conclusion:**

The Holo has the potential to be a clinically useful yet affordable diagnostic tool suitable for the first time of equipping eye care workers in low resource settings with a BIO at volume.

## Introduction

Globally, 553 million people have some degree of visual impairment with an estimated 43.3 million being blind [1]. Nine out of ten of those with blindness live in low- and middle- income countries (LMICs) [1] with 80% being avoidable with early diagnosis and treatment [2]. Hence, the Lancet Commission on Technologies for Global Health and WHO both recommend sustainable and innovative development of frugal diagnostic tools to meet the needs of users in LMICs [3, 4].

The binocular indirect ophthalmoscope (BIO) is an important diagnostic tool for assessing posterior segment eye diseases. The BIO, in contrast to the view achievable with the more commonly available direct ophthalmoscope, offers a wide stereoscopic view of the retina, often despite hazy media [5]. Traditional BIOs are, however, typically expensive, depend on hard to find and costly consumables, are unnecessarily complex to use and often lack the durability needed to withstand use in rural settings. As a consequence they are rarely found outside of major eye units in financially stretched health care services.

**Fig. 1:**
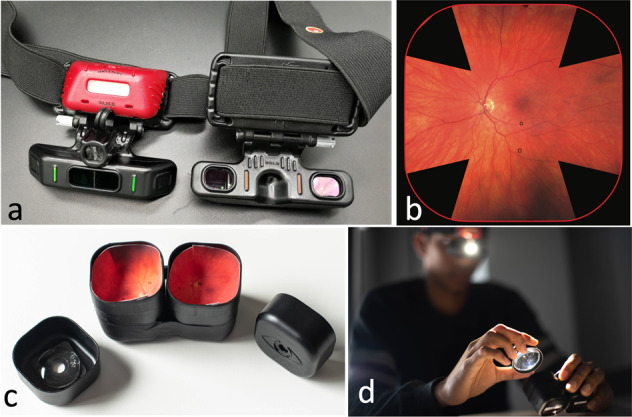
Holo binocular indirect ophthalmoscope and simulation eyes. **a** Frugal ‘Holo’ binocular indirect ophthalmoscope. **b** Lea symbols printed on gored fundi. **c** Hemi-spherical fundi placed in simulation eyes. **d** Participant examining pair of simulation study eye.

Consequently, a low cost solar powered BIO (Fig. 1a and Video 1) called the ‘Holo’ has been developed using a frugal engineering approach [6]. Through use of fixed prism wide-entry eyepieces and a single light emitting diode (LED) illumination system manufacturing costs are minimised and simplicity of use maximised. The Holo is designed to be comfortable to wear with an adjustable elastic band, weighing 100 grams with charge lasting for 4 h when using maximum illumination (Video 1).

In this study we compare the optical quality and design characteristics of the Holo ($75) with a widely available traditional spectacle mounted device, the Keeler Spectra Iris Indirect Ophthalmoscope (Keeler BIO - $3203.90) amongst a range of eye care practitioners.

## Materials and methods

University Teaching and Research Ethics Committees of the University of St Andrews granted approval to undertake the study which followed the tenets of the Declaration of Helsinki.

Twenty-four participants who used a BIO in their daily work were recruited (12 consultant ophthalmologists, 10 ophthalmology residents and 2 hospital optometrists). They examined simulated eyes (SE – Fig. 1b, c) using both the Holo and the Keeler BIO with a 30-diopter Volk condensing lens (Fig. 1d). After a 30 min familiarisation session with the two devices examining training SEs, all participants examined five pairs of study SEs with both devices randomly assigned to start with the either the Holo or the Keeler BIO. Two Lea symbols (apple, square, house or circle) of varying sizes were printed on retinal images at the back of each SE (Fig. 1b) [7]. The participants had one minute to attempt to identify the two Lea symbols printed on the fundus of each SE. The correct identification and time taken to identify the symbols was recorded.

Twelve of the participants additionally examined SEs with Frisby-style stereo plates of two different thicknesses (60 and 90 arcsec) instead of images of fundi.

After using the two BIOs a 7-point Likert scale was used to rate the following characteristics: ease of use, quality of illumination, robustness, portability, comfort of wear, clarity of view, binocularity, field of view and eye strain.

Participants were finally asked how frequently they used the additional features of a BIO including: safety filter, cobalt blue filter, red-free filter and variable aperture using a 5 option response; never and not aware of the feature, never but aware of the feature, less than 50% of the time, half of the time or more, and 100% of the time.

Using IBM SPSS Statistics V.26, paired *t* test was performed for the paired continuous variables. Wilcoxon signed-rank test was performed for the paired ordinal variables. All tests were two-tailed with type I error set at α = 0.05 and it was ensured that data met the particular test’s assumptions.

## Results

There was no statistical difference in the diagnostic and optical performance of the two devices. The number of Lea symbols correctly identified using either device (15.63/20 (Standard deviation (SD) = 3.74) for Holo vs. 15/20 (SD = 3.35) for Keeler BIO, *p* = 0.366, paired *t* test) was statistically the same. There was also no difference in time taken to correctly identify each symbol (Holo 0.39 s faster; 95% confidence interval −2.24 to 3.03 s, *p* = 0.763, paired *t* test, mean time per symbol for Holo = 14.44 s (SD = 6.17) and mean time per symbol for Keeler = 14.83 s (SD = 5.86)).

There was no statistically significant difference between the different participants in either the number of symbols seen or time taken to identify.

12 out of the 12 participants achieved stereoacuity of 60 arcsec using the Holo while with the Keeler BIO 11 achieved 60 arcsec and one 90 arcsec.

There was no statistically significant difference in the scores recorded for clarity of view, quality of illumination, field of view, binocularity, eye strain and robustness between the two devices. The Holo however scored statistically significantly higher for ease of use (6.5 vs. 6, *p* = 0.00488, Wilcoxon signed-rank test), comfort of wear (6 vs. 5, *p* = 0.000337, Wilcoxon signed-rank test) and portability (7 vs. 6, *p* = 0.000148, Wilcoxon signed-rank test).

Most additional features of a traditional BIO are used infrequently with some being rarely used, if at all. The safety filter was almost never used with 2/3 of participants were unaware of the existence of the feature. The most commonly used feature was varying illumination, which was utilised at least half the time by participants. The rate of usage of different features of traditional BIOs has been summarised in Table 1.

**Table 1:** Frequency of additional features of a binocular indirect ophthalmoscope used by the participants in their daily practice.

Features	Safety filter	Cobalt blue filter	Red free filter	Varying light aperture	Varying illumination
Unaware of this feature	67%	0%	0%	0%	0%
Aware of this feature but never use it	25%	38%	54%	17%	0%
Use it <50% of the time	4%	50%	46%	33%	0%
Use it ≥50% of the time	4%	8%	0%	25%	42%
Use it 100% of the time	0%	4%	0%	25%	58%

## Discussion

High resource countries make extensive use of widely available sophisticated and expensive technology while those living in the world’s poorest countries, where the burden of disease is greatest, lack access to even the most basic drugs and equipment [3, 4].

We show the Holo to offer users a comparable view of the posterior segment to that achieved with a more expensive traditional Keeler BIO. This is evidenced by users of either device being equally able to accurately identify symbols of varying size and location printed onto the fundi of SEs. In addition, despite using a fixed IPD viewing arrangement, objective stereoacuity achieved with the simplified BIO design was no different from a device with a variable viewing system.

The equal optical performance of the two devices was further supported by the participants’ opinions on the design characteristics where the clarity of view, quality of illumination, field of view, perception of binocularity, eye strain and robustness of the two devices were found to be equivalent. Users however favoured the Holo for ease of use, comfort and portability compared to the Keeler BIO. However, longer term studies will need to be performed to confirm these findings hold for sustained use.

We used objective non-clinical symbols in simulated eyes rather than real pathologies in patients to create an objective assessment tool that focused on the attributes of the different BIOs rather than being confounded by varied knowledge and experience of users as well as the subjectivity of interpreting real clinical signs. Indeed, there was no difference in performance of the participants despite their range of clinical experience.

Interestingly most of the additional features of traditional BIOs that add to the cost, complexity and likelihood for failure were reported to be infrequently used, if even known about at all. The most used feature was variable illumination, a feature present on the Holo.

The ability to bring the viewing and illumination axis closer to improve the view through small pupils is an optical advantage of traditional devices. This feature is not present in the Holo as a fixed viewing system to reduce the number of moving parts is key to maximising durability and functional longevity. In creating a frugal and durable device these engineering trade-offs are unavoidable. To partly overcome this limitation it is possible to reduce the brightness of the illumination to minimise pupil constriction.

The light source intensity and spectral spread of the Holo is consistent with a Class 1 medical device. We are Currently seeking United Kingdom Conformity Assessed (UKCA) and Conformitè Europëenne (CE) accreditation for this [8, 9]. In addition, a green filter cap for the light source is in development to allow examination of the retinal nerve fibre layer in more detail. With accreditation and these additional features in place studies in a clinical setting can be performed to explore the findings from this initial study involving simulation eyes.

In conclusion, this study demonstrates that the Holo has the potential to be a clinically useful yet affordable diagnostic tool suitable for the first time of equipping eye care workers in LMICs with a BIO at volume. The need to develop frugal technologies using innovative designs and manufacturing techniques to equip healthcare workers in low resource setting is more urgent than ever. The Holo represents such a device that can complement the many other strategies being established globally to reduce avoidable blindness [10].

### Summary

#### What was known before

- Binocular indirect ophthalmoscope is an important device in diagnosing retinal disease.
- Traditional binocular indirect ophthalmoscope is too expensive to be widely available in low and middle income countries.

#### What this study adds

- Holo, a frugal binocular indirect ophthalmoscope, offers users a comparable view of the retina to that achieved with a more expensive traditional binocular indirect ophthalmoscope.
- Holo can be used to equip eye care workers in low resource settings with a binocular indirect ophthalmoscope at volume.

## Supplementary information

Holo – A frugal solar powered binocular indirect ophthalmoscope
